# CITED4 gene silencing in colorectal cancer cells modulates adherens/tight junction gene expression and reduces cell proliferation

**DOI:** 10.1007/s00432-015-2011-5

**Published:** 2015-08-05

**Authors:** Michael A. Rogers, Verena Kalter, Gemma Marcias, Marc Zapatka, Sebastian Barbus, Peter Lichter

**Affiliations:** Division of Molecular Genetics (B060), German Cancer Research Center, Im Neuenheimer Feld 580, 69120 Heidelberg, Germany

**Keywords:** Gene expression, Colorectal cancer, Microarray, Tight junctions, Adherens junctions, c-MET, Actin, Claudins, Ezrin, CITED4

## Abstract

**Purpose:**

CITED4 is one member of a family of transcriptional cofactors, several of which are deregulated in a variety of tumors, including colorectal cancer (CRC). We modulated CITED4 expression, in vitro, and analyzed the associated phenotypic and gene expression changes.

**Methods:**

CITED4-overexpressing and shRNA-mediated knockdown cell lines and control cell lines were established in the CRC cell line SW480. The cells were analyzed for changes in proliferation, apoptosis/cell cycle, migration, invasion, colony formation and adhesion. mRNA expression changes were determined by microarray and pathway analysis, and several deregulated genes were validated by qRT-PCR and Western blotting. Based on results obtained from these studies, the status of the actin cytoskeleton was evaluated by phalloidin/vinculin staining.

**Results:**

Phenotypically, the CITED4-overexpressing cell line showed only moderate changes in adhesion. Microarray analysis identified several deregulated genes, including several G protein-coupled receptors. Phenotypic analysis of the CITED4 shRNA knockdown cell line demonstrated decreased cell proliferation and G2 cell cycle blockage. Microarray analysis identified many deregulated genes, and pathway analysis discovered genes linked to actin-associated adherens junctions/tight junctions (claudin-4, claudin-7, ezrin, MET, ß-catenin). Phenotypically, no morphological changes of the actin cytoskeleton were seen.

**Conclusions:**

Upregulation of CITED4 in SW480 resulted in no obvious phenotype. CITED4 shRNA-mediated knockdown led to decreased cellular proliferation and modulation of a large number of genes, including the c-MET tyrosine kinase and several actin-associated adherens junctions/tight junction genes.

**Electronic supplementary material:**

The online version of this article (doi:10.1007/s00432-015-2011-5) contains supplementary material, which is available to authorized users.

## Introduction

CITED4 (CREB-binding protein/P300-interacting transactivator with ED-rich tail, member 4) is one member of the CITED family of proteins known to participate in the regulatory activity of several genes, including TGFB, ER, TFAP2 and HIF1A. CITED1 (msg1), CITED2 (mrg1, p35srg) and CITED4 (mrg2) were originally identified as a family of homologous proteins possessing a conserved transactivating (CR2) domain that binds and transactivates the transcriptional regulator/histone acetyl transferase CBP/P300. A mouse transcript for CITED4 was originally identified, shown to be expressed in a wide variety of tissues, and to bind CBP/P300 (Yahata et al. 2002). Braganca et al. (2002) successfully identified human CITED4 transcripts, demonstrated its expression in a large number of human organs and showed that the carboxyterminal end of the resulting protein interacts with the CH1 domain of HIF-1alpha and, similar to CITED2 (Bamforth et al. 2001), also interacted with all known human isoforms of the transcription factor AP2 (TFAP2). CITED4 was shown to negatively regulate the binding of HIF-1alpha to CBP/P300, thus inhibiting hypoxia-activated transcription (Fox et al. 2004). Several members of the CITED gene family as well as CBP/P300 have been implicated in carcinogenesis. Analysis of CITED4 in a large series of human breast tumors by immunohistochemistry showed both cytoplasmic and nuclear staining, the nuclear staining of CITED4 negatively correlating with HIF-1alpha expression, tumor size and tumor grade (Fox et al. 2004). In a further study, Tews et al. analyzed tumor material from patients with oligodendral brain tumors exhibiting a loss of chromosomes 1p 19q. Comparison of gene expression in these patients with tumors not possessing the 1p/19q deletions showed downregulation of CITED4 expression (Tews et al. 2006). In a further patient study, DNA methylation and mutation analysis of patients possessing the 1p 19q deletions showed that the CITED4 promoter regions of patients harboring the deletions were often methylated, and this methylation was associated with longer overall/progression-free survival (Tews et al. 2007). No mutations in CITED4 were observed either in this study or in one further analysis of glioblastoma patients (Tews et al. 2007; Torres-Martin et al. 2008). In breast cancer patients, no mutations and only infrequent hypermethylation was observed (Huang et al. 2011).

Our previous efforts at CITED4 analysis in oligodendral brain tumors (Tews et al. 2006, 2007) coupled with previous publications describing a role for another member of the CITED family (CITED2) in colorectal (CRC) cancer invasion (Bai and Merchant 2007), led to this current study which deregulates and analyzes CITED4 activity in a colorectal cancer cell line.

## Methods

### Cell culture

SW480 and HEK293T cells were obtained from the German Collection of Microorganisms and Cell Cultures (DSMZ). Cultures were grown in RPMI-1640 medium (Sigma) containing 10 % fetal bovine serum (FBS) (Biochrom) and 100 u penicillin/100 µg streptomycin (Sigma). All cell lines were passaged every 3–4 days. The cell lines in this study were regularly tested for contamination and authenticity.

### CITED4 overexpression and knockdown vectors

For the overexpression construct, the open reading frame of human CITED4 (nm_133467, 220–774 bp) was amplified by PCR and then cloned into the pCDNA4 HISMAX vector (pCNDA4 HISMAX TOPO TA Expression Kit, Invitrogen). Thereafter, a green fluorescent protein (GFP gene containing an internal ribosomal entry site (IRES-GFP), a kind gift of Dr. Daniel E. Stange, Dresden, Germany) was cloned into the EcoRI-XhoI site of the CITED4 construct as well as into the empty vector, and both constructs (IRES-GFP and CITED4-IRES-GFP) were sequenced. For the knockdown experiments, five short hairpin (sh) RNA constructs to CITED4, as well as a control hairpin, GFP and empty (pLKO.1) vector were obtained from the human RNAi Consortium library (Sigma-Aldrich) (Moffat et al. 2006). Four of the five CITED4 shRNA constructs showed significant knockdown in SW480 cells following transient transfection and qRT-PCR. Two of the shRNAs TRCN0000020921 (CCGGCGCCGAACTCATCGACGAGGACTCGAGTCCTCGTCGATGAGTTCGGCGTTTTT) (ND1) and TRCN0000020923 (CCGGCGGCATGGACGCCGAACTCATCTCGAGATGAGTTCGGCGTCCATGCCGTTTTT) (ND2) were used for the creation of two permanent cell lines. The second shRNA-containing cell line (TRCN0000020293) (ND2) was used for complete phenotypic characterization and microarray analysis.

### Lentivirus preparation

Replication-incompetent lentivirus (CITED4 shRNA, control shRNA or empty vector) was created using a second-generation lentiviral packaging system. The CITED4 shRNA/control plasmid constructs (7 µg) were co-transfected together with the helper plasmids pMD2.G (2.1 µg) and PSPAX2 (5.7 µg) into 2 × 10^6^ HEK293T cells/10 cm plate using Trans-IT-L1 transfection reagent according to the manufacturer’s instructions (Mirius, Madison, WI). The medium was changed after the first day, and the virus-containing medium was harvested 2 days later. The virus particles were concentrated by ultracentrifugation (SW41 rotor, 25,000 RPM, 1.5 h) in a Beckman L70 ultracentrifuge (Beckman-Coulter, Krefeld, Germany) and taken up in 100 µl PBS. The lentiviral titer was determined by GFP transduction in HEK293T cells as well as by puromycin-selected limited dilution and colony formation.

### Preparation of permanent cell lines

The CITED4-overexpressing cell line and control cell line were prepared by transfection of SW480 with the IRES-GFP and CITED4-IRES-GFP constructs using Jetprime transfection reagent (PeqLab, Erlangen, Germany) according to the manufacturer’s recommendations. Selection was performed starting at day 3 using 300 µg/ml zeocin (Invitrogen/Life Technologies, Darmstadt, Germany). For the creation of CITED4 shRNA permanent cell line/control cell lines, SW480 cells were transduced with a tenfold multiplicity of infection (MOI) of the respective lentivirus (see above). The virus was removed by medium change at day 1, and selection was performed starting at day 3 using 2 µg/ml puromycin.

### Proliferation and colony formation assays

Proliferation of both the CITED4-overexpressing/shRNA cell line was analyzed. To this end, 5 × 10^4^ cells of each cell line were plated in triplicate into 12-well plates and grown for a period of 8 days. The wells were harvested at day 2, 4, 6 and 8 and the cells counted using a Vi-Cell XR cell counter (Beckman-Coulter, Krefeld, Germany). For colony formation assays, 1 × 10^3^ cells of each respective cell line were plated in triplicate into six-well plates using 2 ml medium/well. The medium was changed every 3 days. After 17 days, the medium was removed, and the cells were washed in PBS and stained 7 min with 2 % methylene blue/50 % ethanol. Thereafter, the excess stain was removed by H_2_O washings. The colonies (>50 cells) were manually quantified using the ImageJ program.

### Further phenotypic assays

(Migration, invasion, cell cycle analysis, adhesion array assay, See Supplementary Methods).

### Microarray analysis

For microarray analysis of both the SW480 CITED4-overexpressing cell line (CITED4-ires-GFP)/control cell line (ires-GFP) and the CITED4 shRNA permanent cell line/control cell lines, 1 × 10^5^ cells from each line were passaged into 12-well plates containing 1 ml RPMI-1640 plus 10 % FCS and penicillin/streptomycin. The cells were harvested at day 1, 3 and 5 for total RNA preparation (see qRT-PCR analysis). Several separate experiments were performed, and equal amounts of total RNA from each specific time points for each cell line were pooled. RNA integrity (RIN > 8.5) was confirmed using an Agilent Bioanalyzer. Microarray hybridization of each of the samples was performed on Agilent 4 × 44 k arrays according to the manufacturer’s instructions using a two-color system with Universal Human Reference RNA (Agilent, Santa Clara, CA) as a control for each sample. The hybridized microarrays were scanned using an Agilent Microarray Scanner (GS2565CA), and raw data were generated from the scanned images using Agilent Feature Extraction Software. Further processing and normalization (filter vsn) were performed using Chipyard, an in-house developed microarray analysis tool. Venn analysis was performed using the Chipster Microarray Analysis Program (Kallio et al. 2011). Microarray data are available in the ArrayExpress database (www.ebi.ac.uk/arrayexpress) under the accession numbers E-MTAB-2486 and E-MTAB-2487.

### R2 analysis and functional gene annotation

Expression analysis of CITED4 using the data of Jorissen et al. (2009) was evaluated using the R2 microarray analysis and visualization platform (http://r2.amc.nl). Functional annotation of the expression data from the microarray analysis obtained in the current study was performed using the DAVID bioinformatics resource (Huang et al. 2009a, b). The DAVID Functional Gene Classification Tool allowed the classification of the deregulated genes into functional groups using key terms associated with each group. In addition, KEGG database analysis grouped relevant genes into specific biochemical pathways. The gene lists generated from these groups were used to identify candidates for further evaluation.

### Microarray candidate gene validation by qRT-PCR

All qRT-PCR experiments were analyzed in accordance with the MIQE guidelines (Bustin et al. 2009). For each sample, 2.2 × 10^6^ of each cell line was plated into 10 cm plates in RPMI-1640 plus 10 % FCS and penicillin/streptomycin and grown for 1, 3 or 5 days. The cells were then harvested for total RNA preparation using the RNEasy kit (Qiagen, Hilden, Germany). Genomic DNA removal and first-strand synthesis (1 µg total RNA) were performed using the Quantitect Reverse Transcription Kit (Qiagen, Hilden, Germany) according to the manufacturer’s instructions. The 20 µl first-strand syntheses were diluted 1/3 in water and 2 µl was used for quantitative real-time PCR together with 10 pmol of the appropriate primer pairs (see Supplementary Table 1) and 6 µl Absolute SYBR Green Rox Mix (Thermo Scientific, Schwerte, Germany). The reaction mixtures were amplified/quantified using the 7900HT Real-Time PCR System thermocycler (Life Technologies, Darmstadt, Germany) using the following cycling conditions 50 °C—2 min, 95 °C—15 min, 40 cycles of 95 °C—15 s, 60 °C—1 min for amplification followed by 95 °C—15 s, 60 °C—15 s for annealing curve determination. Standard curves were performed for each of the amplifications. The genes of interest were normalized against the housekeeping genes DCTN2 and PGK1.

### Western blot analysis

Cell were harvested, pelleted and resuspended in 3× amount of RIPA buffer (50 mM Tris/HCL pH7.4, 150 mM NaCl, 1 % Triton X100, 0.5 % deoxycholate, 0.1 % SDS) with protease inhibitors (Complete Mini, Roche), incubated on ice for 45 min and pelleted thereafter by centrifugation. The supernatant was quantitated using BCA protein detection reagent (Pierce, Rockford, IL). Twenty micrograms of the protein lysate was separated on 4–12 % polyacrylamide gels (Nupage-Novex, Invitrogen/Life Technologies, Darmstadt, Germany) using 1× MES-SDS as a running buffer. The separated proteins were transferred electrophoretically (2 h at 240 mAmp) onto a PVDF membrane (Millipore, Billerica, MA) using an XCELLII transfer module (Invitrogen) in transfer buffer (48 mM Tris, 39 mM glycine, 20 % methanol). Following the protein transfer, the blots were incubated in blocking buffer [5 % milk powder in TBS (50 mM Tris pH7.4, 150 mM NaCl)] for 1 h. Thereafter, the blot was incubated with the primary antibody at the appropriate dilution (see Supplementary Table 2) in blocking buffer for 1 h or overnight. Then, the antibody was removed and the blot washed 3 × 10 min in TBST (TBS with 0.02 % Tween 20). Thereafter, the blot was incubated with the secondary antibody at the appropriate concentration (see Supplementary Table 2) in blocking buffer for 1 h. After three further washes in TBST, the protein of interest was identified using enhanced chemiluminescence (ECL, Pierce) coupled with X-ray film exposure.

### Actin/Focal adhesion assay

2 × 10^5^ cells of the CITED4 knockdown cell lines and the respective controls were passaged onto sterile Labtek Chamber slides (Brand, Wertheim, Germany) in 1 ml RPMI-1640 plus 10 % FCS and penicillin/streptomycin. The slides were prepared for fluorescence microscopy of actin (phalloidin-TRIC staining) and vinculin (Alexa 488 staining) using an actin and focal adhesion staining kit (Millipore) according to the manufacturer’s instructions. The dilutions of phalloidin-TRIC, vinculin primary antibody and the secondary antibody can be found in Supplementary Table 2.

### Statistical analysis

Statistical evaluation of the phenotypic data/qRT-PCR assays were demonstrated by calculation of standard deviation for small samples (*n* − 1) or two-tailed Student’s *t* tests on 2–3 separate experiments with a total sample size of 6–9. The exact number of experiments and sample size are shown in the figure legends.

## Results

The R2 database, a collection of previously published high-content molecular studies (R2: microarray analysis and visualization platform-http://r2.amc.nl), was screened for data of CITED4 in CRC. Microarray expression data from 226 patients exhibiting different stages of CRC (Jorissen et al. 2009) were evaluated through the R2 database by Kaplan–Meier analysis. Fourteen percent of the patients in the study exhibited a high CITED4 expression level which was associated with a tendency toward poorer relapse-free survival (Supplementary Fig. 1).

**Fig. 1:**
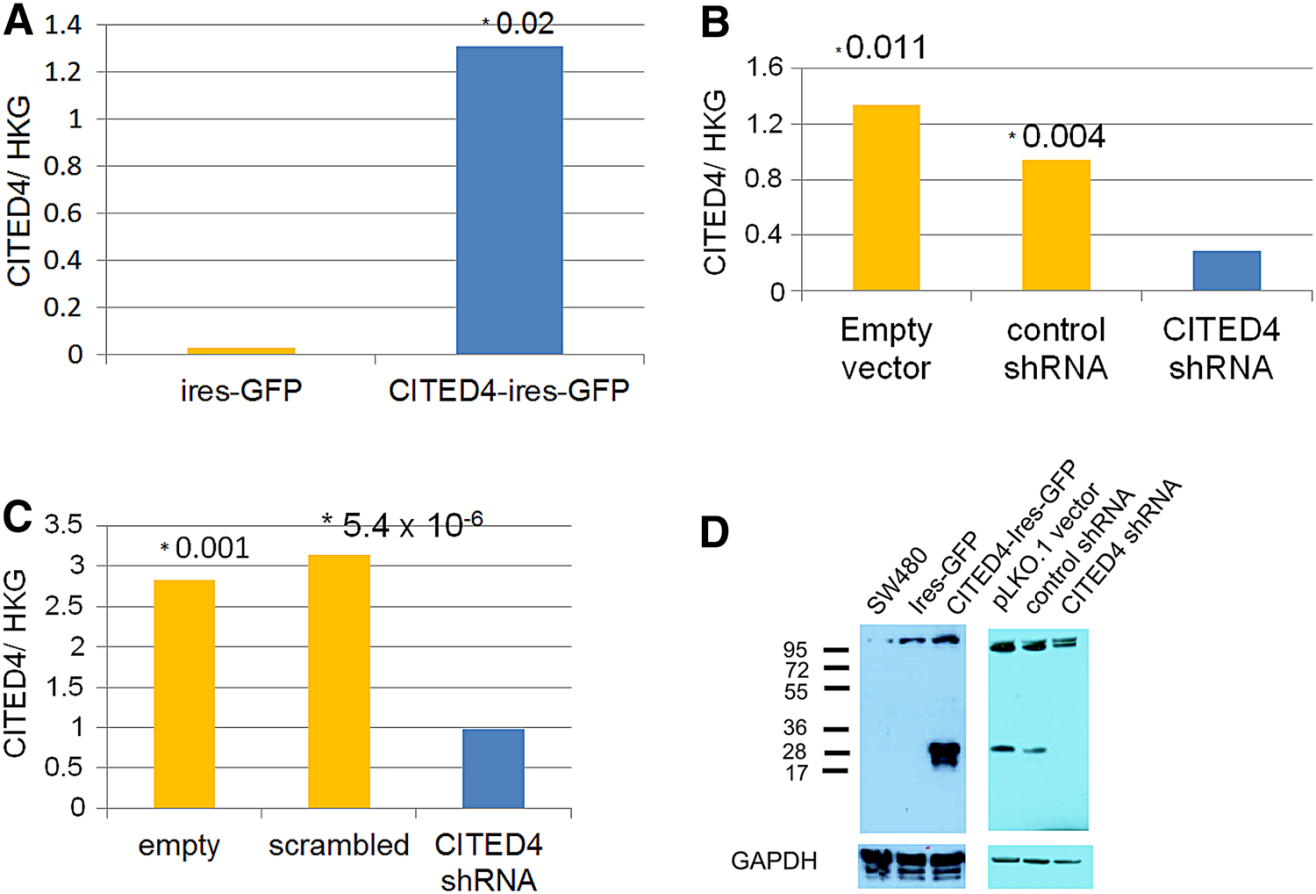
Validation of CITED4 expression in the SW480 CITED4-overexpressing/shRNA-mediated knockdown permanent cell lines **a**–**c** qRT-PCR analysis of the respective cell line at day 3 (three experiments, *n* = 6). **a** CITED4-overexpressing cell line. **b** First CITED4 shRNA permanent cell line. **c** Second CITED4 shRNA permanent cell line. **d** Western blot analysis of the CITED4-overexpressing permanent and control cell line (*left*); second CITED4 shRNA permanent cell line and controls (*right*). *, *t* test

**Fig. 2:**
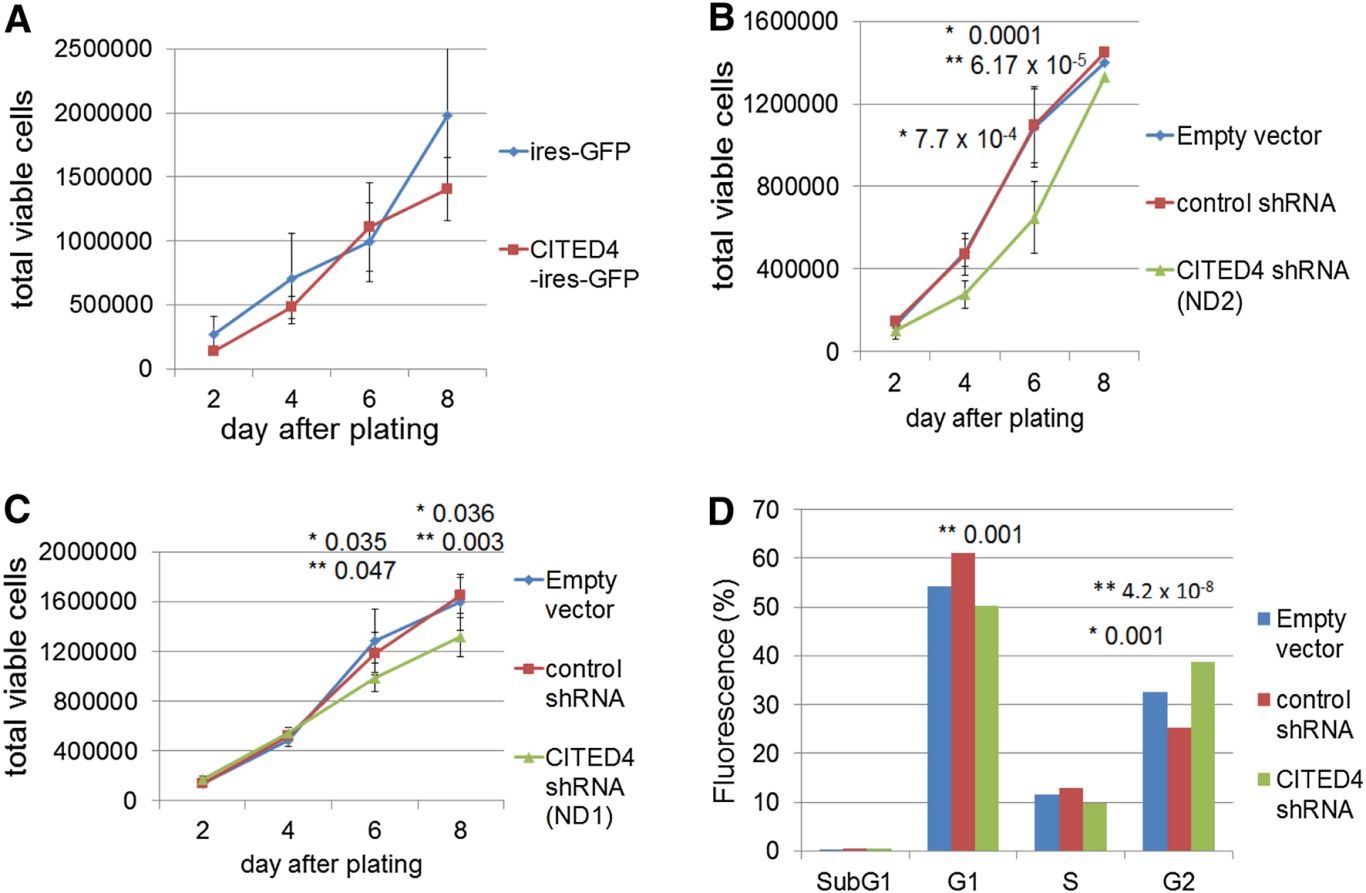
Proliferation and cell cycle analyses of the CITED4-overexpressing/shRNA permanent cell lines. **a**–**c** Time course of proliferation for the respective cell lines (two experiments, *n* = 6). **a** Proliferation assay—CITED4-overexpressing cell line and control. **b**, **c** Proliferation assay—first (ND1) and second (ND2) CITED4 shRNA permanent cell lines. **d** Quantitation of the histograms (two experiments, *n* = 6). *t test, empty vector versus CITED4; **t test control shRNA versus CITED4

In order to analyze the effects of CITED4, in vitro, the CRC cell line SW480, derived initially from a 50-year-old male with Dukes stage B CRC (Leibovitz et al. 1976), was used to generate both a CITED4-overexpressing and two CITED4 shRNA-mediated knockdown permanent cell lines. qRT-PCR (Fig. 1a–c) and Western blot analysis (ND2, Fig. 1d) of both cell lines showed a strong upregulation of CITED4 (ca. 20 fold by qRT-PCR—Fig. 1a) for the overexpressing cell line and a strong downregulation (ca 80–90 %) in the two shRNA knockdown cell lines (Fig. 1b, c). Western blot analysis of the CITED4-overexpressing cell line showed a strong increase in CITED4 protein production (Fig. 1d, left side). Similar analysis in the ND2 CITED4 knockdown cell line showed the complete ablation of protein production (Fig. 1d, right side; Note: The following experiments were performed with the ND2 knockdown cell line due to its better degree of downregulation). The phenotype of both the overexpressing and the second shRNA knockdown cell lines were evaluated by proliferation studies, cell cycle analyses, clonogenicity assays, migration (scratch), invasion assays as well as adhesiveness to several basement membrane proteins. While the CITED4-overexpressing cell line showed no difference in proliferation when compared to the control cell line (Fig. 2a), both of the CITED4 shRNA knockdown cell lines (ND1 and ND2) showed a decrease in cell proliferation (Fig. 2b, c), and the second cell line showed a much stronger decrease in proliferation (ca. 40 %) during the linear growth phase when compared to the empty vector and control shRNA cell lines (Fig. 2b). Therefore, the ND2 CITED4 shRNA knockdown cell line was used for all further analyses. Cell cycle analysis of the CITED4 shRNA knockdown cell line at day 4 after plating (Fig. 2d) showed no change in the subG1 fraction of the cell cycle when compared to the empty and control vectors, indicating that the inhibition of proliferation was not associated with apoptosis. Additional assessment of apoptosis was also evaluated in these cells lines by FACS analysis of annexin V/7-AAD stainings, but these experiments showed no changes in annexin V levels or cell viability when compared to controls (data not shown). The cell cycle analyses did show, however, a moderate increase in the G2 fraction when compared to the control cell lines, indicating that the inhibition of proliferation was associated with a moderate G2 blockage of the cell cycle (Fig. 2d).

Clonogenicity studies of both the CITED4-overexpressing and CITED4 shRNA permanent cell line showed no changes in proliferative capacity between the CITED4 deregulated cell lines when compared to the respective control lines (Supplementary Fig. 2A–C).

Migration (scratch) assays of both the overexpressing and knockdown cell line were analyzed at 0, 6, 12, 24 and 30 h after scratching (only the 30 h analysis is shown, Supplementary Fig. 3). No change in migratory activity was seen between the overexpression/and shRNA knockdown cell lines when compared to the control cell lines. Cellular invasion was also analyzed in the CITED4-overexpressing/shRNA knockdown cell lines using Matrigel-coated Transwell chambers at 48 h after plating (Supplementary Fig. 4). No increase in invasiveness was seen in the CITED4-overexpressing cell line (supplementary Fig. 4A), and a tendency toward increased invasiveness seen in the CITED4 shRNA permanent cell line was not statistically significant (Supplementary Fig. 4B).

Analysis of adhesiveness to several known basement membrane proteins using a commercially available adhesion array system demonstrated an increased adhesiveness of the CITED4-overexpressing cell line to collagen IV and vitronectin when compared to the control cell line (Supplementary Fig. 5A). In contrast, no changes in adhesiveness to any of the basement membrane proteins were seen in the CITED4 shRNA knockdown cell line compared to controls (Supplementary Fig. 5B).

**Fig. 3:**
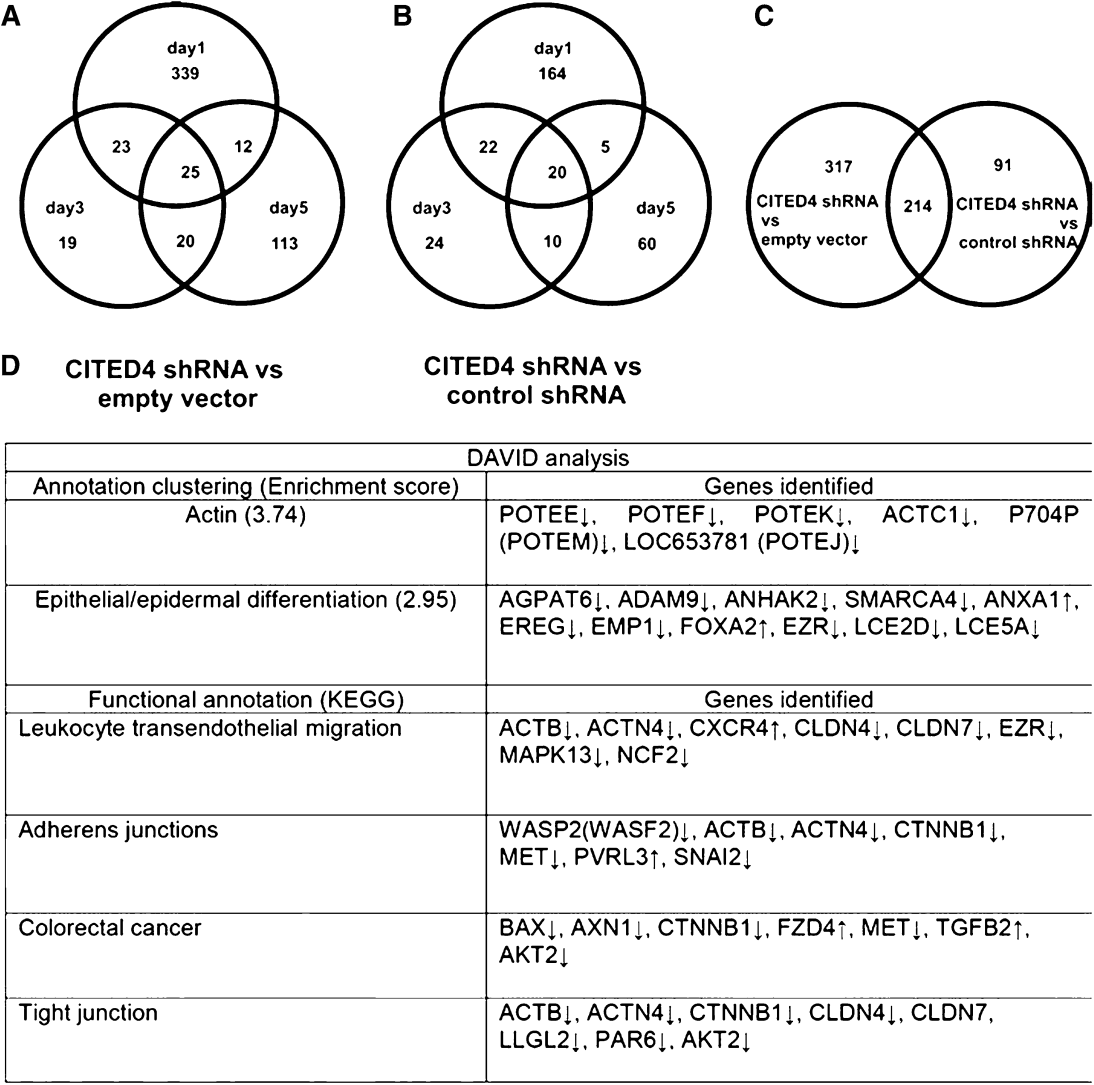
Venn diagram of the CITED4 shRNA permanent cell line microarray data and DAVID pathway analyses. **a** CITED4 shRNA/empty vector, day 1, 3, 5. **b** CITED4 shRNA/control shRNAs, day 1, 3, 5. **c** Total CITED4 shRNA/empty vector versus CITED4 shRNA/control shRNA. **d** Results of the DAVID pathway analysis

Expression analysis was performed in the CITED4-overexpressing, as well as the CITED4 shRNA knockdown cell lines, at day 1, 3 and 5 after plating using Agilent 4 × 44 K microarrays. Upon comparison of the overexpressing cell line with the control line, very few genes (120) were deregulated over the 5-day period, despite using a log2 fold change of one as a cutoff for deregulation (see Supplementary Table 3). In contrast, analysis of the CITED4 shRNA permanent cell line compared to the empty vector/control shRNA cell lines showed a larger number of deregulated genes (551 CITED4 shRNA/empty vector; 306 CITED4 shRNA/control shRNA) (Fig. 3a–c, Supplementary Table 4), 214 of which were present in both datasets. Using the significantly deregulated datasets from the CITED4-overexpressing/knockdown cell lines, functional annotation analysis was performed using the DAVID bioinformatics resource. In the CITED4-overexpressing cell line dataset, no obvious pathways were discerned from the data. However, manual annotation and literature search demonstrated that, in addition to SYK kinase, three G protein-coupled receptors were deregulated (LGR6, GPR64, GPR110), two of which are, by homology, adhesion-associated G protein-coupled receptors. The DAVID database identified two putative annotation clusters in the CITED4 shRNA permanent cell line (actin, epithelial/epidermal differentiation, see Fig. 3d). Functional analysis using the Kyoto Encyclopedia of Genes and Genomes (KEGG) pathway database integrated into the DAVID program identified four functionally annotated cellular processes, namely leukocyte transendoethelial migration, adherens junctions, colorectal cancer and tight junctions. Many of the genes described in these pathways overlapped with each other (Fig. 3d).

**Fig. 4:**
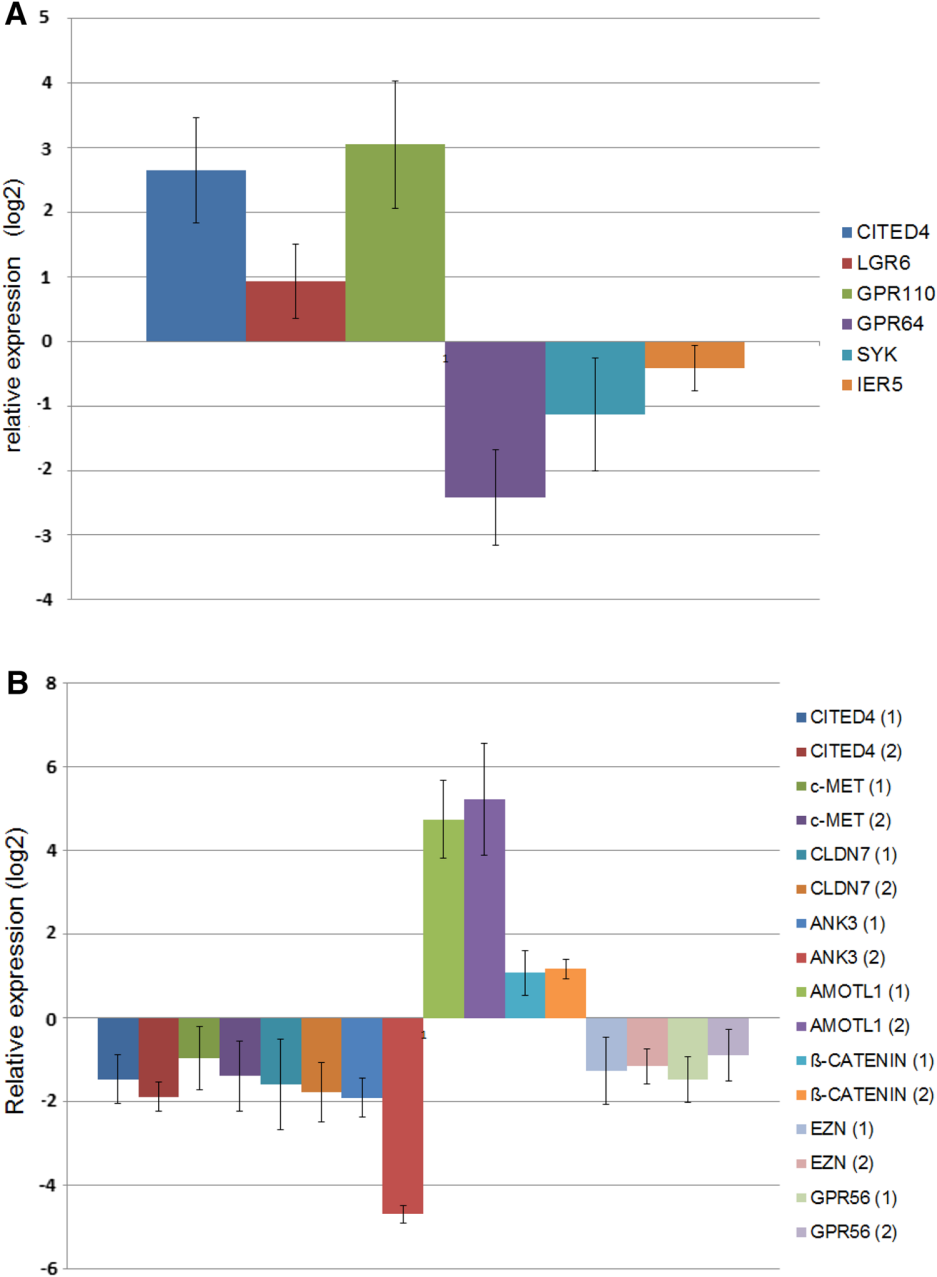
qRT-PCR validation of a subset of deregulated genes identified from CITED4-overexpressing/shRNA knockdown permanent cell line microarray analysis. Analyses performed at day 5 after plating (three experiments, *n* = 6). **a** CITED4-overexpressing cell line/empty vector cell line, **b** CITED4 shRNA permanent cell line, *1* CITED4 shRNA cell line/empty vector cell line, *2* CITED4 shRNA cell line/control shRNA cell line

**Fig. 5:**
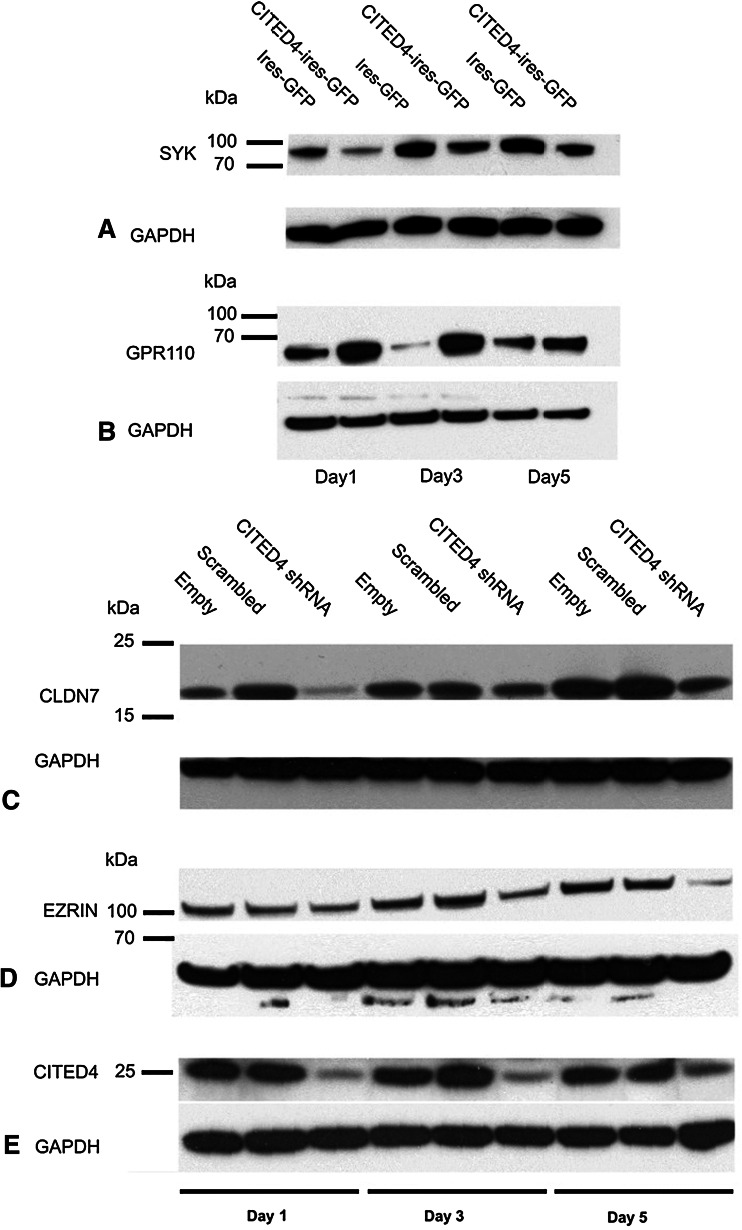
Western blot validation for a subset of deregulated genes derived from the CITED4-overexpressing/shRNA knockdown cell line microarray analyses. **a** SYK, **b** GPR110, **c** claudin-7 (CLDN7), **d** ezrin (EZR). GAPDH was measured as an internal standard

In order to validate our CITED4-overexpressing/knockdown microarray data, we performed time courses for quantitative real-time PCR (qRT-PCR) analysis on several genes derived from either the manual search of the overexpressing cell line or, for the knockdown cell line, the KEGG pathway genes list using RNA derived from multiple time course experiments. Although nearly all of the genes analyzed showed a tendency to correlate with the microarray data in general, only ca. 50 % of the genes analyzed showed a statistically significant deregulation, and a great majority were deregulated at more than one time point. Figure 4a, b shows the day 5 values of the significantly deregulated genes. Three of the 13 genes shown to be significantly deregulated by qRT-PCR were also validated at the protein level by Western blot analysis (Fig. 5).

In order to see if the knockdown of CITED4 in the shRNA permanent cell line induced obvious morphological cell changes in either the actin cytoskeleton or in focal adhesion contact points, the CITED4 shRNA permanent cell line and controls were stained with phalloidin (F-actin) and vinculin (focal adhesions) 3 days after cell line plating (Supplementary Fig. 6). No obvious changes in either the actin filament cytoskeleton or in focal adhesion contact points were seen at this time point.

Downregulation of c-MET might be causal for the inhibition of proliferation seen in the CITED4 shRNA permanent cell line. As such, transient transfections of the CITED4 shRNA permanent cell lines were performed using a c-MET overexpression construct in order to rescue the phenotype. Overexpression of c-MET resulted in a strong increase in c-MET mRNA and protein expression in the CITED4 shRNA permanent cell line as well as in the control cell lines, but the c-MET overexpression was not accompanied by increased proliferation in any of the cell lines (data not shown).

The effect seen in the SW480 CITED4 shRNA permanent cell line (ND2) encouraged us to create two further CRC permanent cell lines by transduction of the same shRNA into LS174T and CL34 cells. Both permanent cell lines exhibited an effective knockdown of CITED4 mRNA. While no inhibition of proliferation was seen in LS174T, the inhibition of proliferation in CL34 was significant. However, as this effect was minor, we decided not to continue evaluation of these cell lines.

## Discussion

Overexpression of CITED4 results in no significant changes in proliferation or colony formation, but shows a moderate upregulation of adhesiveness to the basement membrane proteins collagen IV and vitronectin, which, however, is not associated with a change in invasive phenotype. Decreased collagen IV expression is seen in CRC patients and is associated with poor prognosis (Havenith et al. 1988; Zeng et al. 1999). Vitronectin is absent in the normal colon, but appears to be expressed in CRC, possibly by induction of the surrounding fibroblasts by the tumor cells (Tomasini-Johansson et al. 1994). CITED4 overexpression modulates expression changes in a small number of genes including three G protein-coupled receptors, GPR64, GPR110 and LGR6. GPR64 and GPR110 are assumed to be adhesion-associated G protein-coupled receptors based on the similarity of their extracellular domains to adhesion molecule-binding domains (Bjarnadottir et al. 2004, 2007). In the epididymis, GPR64 co-localizes with actin and ezrin, which were also identified in the CITED4 shRNA knockdown microarray studies (see below). GPR110 has been shown to be overexpressed in lung and prostate cancer (Lum et al. 2010) and is upregulated in the CITED4-overexpressing cell line. LGR6, a stem cell-associated G protein-coupled receptor modulated by the R-spondin family of ligands (Gong et al. 2012), exhibits mutations and hypermethylation in a subset of analyzed CRC patients (Sjoblom et al. 2006; Schuebel et al. 2007). One further gene of interest is the SYK tyrosine kinase. Although primarily studied in lymphoid cells, numerous solid cancers have been shown to exhibit SYK deregulation, and SYK has been implicated as a putative tumor suppressor gene (Coopman and Mueller 2006). SYK overexpression in CRC cell lines leads to decreased proliferation and invasiveness and appears to be hypermethylated in a subset of CRCs (Yang et al. 2013).

Downregulation of CITED4 results in a decrease in cell proliferation in the SW480 cell line coupled with a moderate blockage in G2 of the cell cycle. No changes in apoptosis [cell cycle subG1 fraction or annexin V/7-AAD analysis (data not shown)], colony formation, migration, invasion or adhesion were noted. Dowregulation of CITED4 in two further CRC cell lines showed significant, but only minor, inhibition of cell proliferation in one of the lines. This is best explained by the inhibition of proliferation seen in the CITED4 knockdown being dependent upon the genetic background in which it occurs.

Knockdown of CITED4 in SW480 cells resulted in the deregulation of a large number of genes, several of which encode proteins seen together with actin-associated adherens/tight junctions (claudin-4, claudin-7, ß-catenin, ezrin) and/or cell proliferation (ß-catenin, MET). In the CITED4 knockdown cell line, downregulation of MET was initially thought to be causal for the inhibition of proliferation seen upon knockdown, for MET and its ligand, hepatocyte growth factor/scatter factor (HGF/SF) is known to influence proliferation in a number of different cell types (Birchmeier et al. 2003; Jiang et al. 1999). In addition, two monoclonal antibodies generated against MET inhibited CRC tumor growth in a mouse xenograft model (van der Horst et al. 2009). However, in the current study, overexpression of c-MET did not lead to an increase in proliferation in the CITED4 shRNA permanent cell line or in SW480, in general (data not shown). C-MET is interesting for several further reasons. c-MET overexpression has been shown to be a prognostic marker in CRC, summarized recently in two clinical study meta-analyses (Gao et al. 2015; Liu et al. 2015 and the references therein). A complex of MET, CD44 variant 6 and the MET ligand HGF has been identified in the colorectal cancer cell line HT-29 and has been demonstrated as being essential for MET activation (Orian-Rousseau et al. 2002). The carboxyterminal portion of CD44 variant 6, which contains a binding region for the actin-associated protein ezrin, was also determined as being necessary for activation. Furthermore, claudin-7, CD44 variant 6, EpCAM and the tetraspanin CP-029 appear to be upregulated in colorectal cancer with liver metastasis and form a common complex in regions termed tetraspanin-enriched membrane microdomains (TEM) (Kuhn et al. 2007). Most recently, EpCAM, CD44 and MET have also been identified in circulating tumor cells (CTCs) of breast cancer patients exhibiting metastasis (Baccelli et al. 2013). Therefore, in the current study, MET could also be a member of a somewhat larger complex, and this complex might be, at least partially, regulated by CITED4. MET is also activated by HIF-1alpha, one of the known binding partners of CITED4 (Pennacchietti et al. 2003). However, CITED4 (as well as CITED2) have been shown to bind and inactivate HIF-1alpha, and in breast tumors, a negative correlation exists between CITED4 and HIF-1alpha protein expression (Huang et al. 2011; Fox et al. 2004; Bhattacharya et al. 1999; Freedman et al. 2003), which would apparently contradict a role for HIF-alpha regulation of c-MET in the current study.

Downregulation of CITED4 modulates the transcription of several proteins associated with adherens/tight junctions (ezrin, claudin 4, claudin 7, ß-catenin, MET) in SW480 cells. Ezrin, a known cytoskeletal linker which binds both to actin filaments and CD44 (Bretscher et al. 2002), is necessary for the maintenance of intestinal villus apical polarity (Saotome et al. 2004), has been shown to be a prognostic marker in a larger clinical study (Patara et al. 2011) and has been implicated, together with CD44, in metastasis (Martin et al. 2003). Claudins are a large group of membranous proteins essential for the formation of tight junctions, multi-protein membrane-associated cellular structures responsible for the maintenance of epithelial cell polarity through isolation and division of the apical and basolateral regions by these structures. Claudin-4 and claudin-7 are known to be important for this polarity. In addition, several claudins also regulate the pericellular passage of small molecules and ions from the epithelial lumen to the underlying tissues (Hartsock and Nelson 2008; Turksen and Troy 2011). In mice, ablation of claudin-7 results in death due to barrier loss with resulting dehydration (Tatum et al. 2010). Analysis of claudins in several different tumor entities shows, with perhaps the exception of claudin 1, no clear-cut pattern between claudin expression and tumorigenicity. Claudin-4 has been shown to be strongly deregulated in gastric cancer (Reviewed in, Turksen and Troy 2011). In CRC, Ueda et al. (2007) showed that primary tumors exhibited similar claudin-4 expression as normal tissue, but invasiveness was associated with decreased claudin-4 expression. In contrast, Mees et al. (2009) saw increased claudin-4 expression in patients with ulcerative colitis-associated CRC. In colorectal cancer, the correlation between claudin-7 expression and metastasis is also not clear. Initially, two studies found that both primary CRC, as well as metastases, showed overexpression of claudin-7, the metastases in one study showing somewhat lower expression compared to the primary tumor (Darido et al. 2008; Kuhn et al. 2007). Kuhn et al. (2007), a clinical study of 102 primary tumors and 66 metastases, also demonstrated co-localization of claudin-7 together with the membrane proteins EpCAM, CD44v6 and the tetraspanin CP-029 and found that this complex is associated with poorer disease-free survival. In contrast, tumor-associated claudin-7 was either unchanged or decreased in invasive areas in three further CRC studies (Nakayama et al. 2008; Oshima et al. 2008; Bornholdt et al. 2011).

CITED4 is one of the three members of the CITED family in humans (CITED1, CITED2, CITED4). The CITED family members show not only fairly high sequence conservation, but also show similar binding partners, such as HIF-1alpha, CBP/P300 and TFAP2. All three members of the CITED4 family have been shown to be involved, to some degree, in tumorigenesis (Meniel et al. 2013; Nasu et al. 2013; Huang et al. 2011; Fox et al. 2004; Tews et al. 2007; Bai and Merchant 2007). In colorectal cancer, CITED1 is overexpressed in human and mouse (APC^min/+^ mouse model) colorectal tumors. Knockdown of CITED1 in the APC^min/+^ mouse model is associated with increased survival accompanied by a paradoxical WNT-associated increase in apoptosis (Meniel et al. 2013; Nasu et al. 2013). Currently, no clinical studies of CITED2 in CRC are available, although CITED2 protein expression is increased in ulcerative colitis, a condition often associated with CRC (Yoshida et al. 2011). shRNA-mediated downregulation of CITED2 in the CRC cell line RKO resulted in a cell morphology change which, by phalloidin staining, was shown to affect the actin cytoskeleton, and this knockdown of CITED2 resulted in increased cellular invasiveness in a Matrigel invasion assay (Bai and Merchant 2007). In addition, microarray analysis was performed in this study, and although the microarray data were not directly presented, the authors identified an increased expression of matrix metalloproteinase-13 (MMP-13), which would tend to support the invasion assay data. As such, this data pointed to a role of CITED2 in CRC invasiveness. In the current study, expression microarray analysis followed by pathway analysis also identified several genes (claudin-7, ezrin, ß-catenin, c-MET) which are known to bind/associate with the actin cytoskeleton. However, neither Matrigel invasion assays nor phalloidin (F-actin) staining was associated with changes in invasiveness or actin cytoskeleton upon knockdown of CITED4, in contrast to what was seen in the CITED2 study. The CITED4 shRNA-mediated knockdown did, however, result in an inhibition of cellular proliferation, which was not seen in the CITED2 study.

## Conclusions

Increased expression of CITED4 in SW480 cells did not lead to an obvious cellular phenotype, but did result in the expression of several (adhesion) associated G protein-coupled receptors. Knockdown of CITED4 in the same cell line inhibited cellular proliferation accompanied by a moderate (G2) cell cycle blockage. Expression analysis showed the deregulation of a large number of genes, including genes involved in the formation of actin-associated adherens/tight junctions, however, without an obvious disruption of the actin cytoskeleton.

## Electronic supplementary material

Below is the link to the electronic supplementary material.

Supplementary Fig. 1. Kaplan-Meier analysis of the data from Jorissen et al (Jorissen et al. 2009) obtained from the R2 database. Blue: patients with higher CITED4 expression; Red: patients with lower expression. Tick marks indicate patient censoring. (JPEG 68 kb)

Supplementary Fig. 2. Colony forming assays CITED4 overexpressing/shRNA knockdown cell lines. A Representative plates taken from the CITED4 shRNA analysis. B-C Evaluation of CITED4 overexpressing-B and shRNA knockdown C cell lines (2 experiments, n = 6).  (JPEG 86 kb)

Supplementary Fig. 3. Migration (scratch) assays-CITED4 overexpressing- (left) and shRNA-knockdown (right) cell line at 30 h after scratching. Two experiments, n = 6.  (JPEG 80 kb)

Supplementary Fig. 4. Invasion assays. A CITED4 overexpressing permanent cell line and control. B CITED4 shRNA knockdown permanent cell line and controls. Invasion was measured 40 h after plating into transwells. *, *t* test values CITED4 overexpressing/shRNA knockdown cell lines vs. controls. Two experiments, n = 6.  (JPEG 109 kb)

Supplementary Fig. 5. Adhesion array analysis. A CITED4 overexpressing permanent cell line and control.  B CITED4 shRNA knockdown permanent cell line and controls. *, Significant *t* test values (*p* < 0.05) CITED4 overexpressing/shRNA knockdown cell lines vs control. Two experiments, n = 6  (JPEG 150 kb)

Supplementary Fig. 6. F-actin (phalloidin) and vinculin immunofluorescence microscopy of the CITED4 shRNA permanent cell line and controls. A empty vector permanent cell line. B control shRNA permanent cell line. C CITED4 shRNA permanent cell line. Orange: actin (phalloidin) staining, green: vinculin staining, blue: DAPI. All pictures at 400x enlargement.  (JPEG 99 kb)

Supplementary methods. (PDF 182 kb)

Supplementary Table 1. Oligonucleotide primers for cloning and qRT-PCR analysis. (PDF 215 kb)

Supplementary Table 2. Antibodies used for western blot analysis and fluorescence microscopy. (PDF 181 kb)

Supplementary Table 3. Significantly deregulated genes-CITED4 overexpressing permanent cell line. Note: Several genes are represented by more than one oligonucleotide on the microarray.  (XLSX 95 kb)

Supplementary Table 4. Significantly deregulated genes-CITED4 shRNA-knockdown permanent cell line. Note: Several genes have more than one oligonucleotide on the microarray. Therefore, the number of positions (clone numbers) in Supplementary Table 4 is larger than the number of genes illustrated in the Venn diagram (Fig. 3).  (XLSX 23 kb)

## References

[CR1] Baccelli I, Schneeweiss A, Riethdorf S, Stenzinger A, Schillert A, Vogel V, Klein C, Saini M, Bauerle T, Wallwiener M, Holland-Letz T, Hofner T, Sprick M, Scharpff M, Marme F, Sinn HP, Pantel K, Weichert W, Trumpp A (2013) Identification of a population of blood circulating tumor cells from breast cancer patients that initiates metastasis in a xenograft assay. Nat Biotechnol 31:539–544 23609047 10.1038/nbt.2576

[CR2] Bai L, Merchant JL (2007) Role for CITED2, a CBP/p300 interacting protein, in colon cancer cell invasion. FEBS Lett 581:5904–5910 18054336 10.1016/j.febslet.2007.11.072 PMC2211427

[CR3] Bamforth SD, Braganca J, Eloranta JJ, Murdoch JN, Marques FIR, Kranc KR, Farza H, Henderson DJ, Hurst HC, Bhattacharya S (2001) Cardiac malformations, adrenal agenesis, neural crest defects and exencephaly in mice lacking Cited2, a new Tfap2 co-activator. Nat Genet 29:469–474 11694877 10.1038/ng768

[CR4] Bhattacharya S, Michels CL, Leung MK, Arany ZP, Kung AL, Livingston DM (1999) Functional role of p35srj, a novel p300/CBP binding protein, during transactivation by HIF-1. Genes Dev 13:64–75 9887100 10.1101/gad.13.1.64 PMC316375

[CR5] Birchmeier C, Birchmeier W, Gherardi E, Vande Woude GF (2003) Met, metastasis, motility and more. Nat Rev Mol Cell Biol 4:915–925 14685170 10.1038/nrm1261

[CR6] Bjarnadottir TK, Fredriksson R, Hoglund PJ, Gloriam DE, Lagerstrom MC, Schioth HB (2004) The human and mouse repertoire of the adhesion family of G-protein-coupled receptors. Genomics 84:23–33 15203201 10.1016/j.ygeno.2003.12.004

[CR7] Bjarnadottir TK, Fredriksson R, Schioth HB (2007) The Adhesion GPCRs: a unique family of G protein-coupled receptors with important roles in both central and peripheral tissues. Cell Mol Life Sci 64:2104–2119 17502995 10.1007/s00018-007-7067-1 PMC11138446

[CR8] Bornholdt J, Friis S, Godiksen S, Poulsen SS, Santoni-Rugiu E, Bisgaard HC, Lothe IM, Ikdahl T, Tveit KM, Johnson E, Kure EH, Vogel LK (2011) The level of claudin-7 is reduced as an early event in colorectal carcinogenesis. BMC Cancer 11:65 21310043 10.1186/1471-2407-11-65 PMC3045986

[CR9] Braganca J, Swingler T, Marques FIR, Jones T, Eloranta JJ, Hurst HC, Shioda T, Bhattacharya S (2002) Human CREB-binding protein/p300-interacting transactivator with ED-rich tail (CITED) 4, a new member of the CITED family, functions as a co-activator for transcription factor AP-2. J Biol Chem 277:8559–8565 11744733 10.1074/jbc.M110850200

[CR10] Bretscher A, Edwards K, Fehon RG (2002) ERM proteins and merlin: integrators at the cell cortex. Nat Rev Mol Cell Biol 3:586–599 12154370 10.1038/nrm882

[CR11] Bustin SA, Benes V, Garson JA, Hellemans J, Huggett J, Kubista M, Mueller R, Nolan T, Pfaffl MW, Shipley GL, Vandesompele J, Wittwer CT (2009) The MIQE guidelines: minimum information for publication of quantitative real-time PCR experiments. Clin Chem 55:611–622 19246619 10.1373/clinchem.2008.112797

[CR12] Coopman PJ, Mueller SC (2006) The Syk tyrosine kinase: a new negative regulator in tumor growth and progression. Cancer Lett 241:159–173 16442709 10.1016/j.canlet.2005.11.004

[CR13] Darido C, Buchert M, Pannequin J, Bastide P, Zalzali H, Mantamadiotis T, Bourgaux JF, Garambois V, Jay P, Blache P, Joubert D, Hollande F (2008) Defective claudin-7 regulation by Tcf-4 and Sox-9 disrupts the polarity and increases the tumorigenicity of colorectal cancer cells. Cancer Res 68:4258–4268 18519685 10.1158/0008-5472.CAN-07-5805

[CR14] Fox SB, Braganca J, Turley H, Campo L, Han C, Gatter KC, Bhattacharya S, Harris AL (2004) CITED4 inhibits hypoxia-activated transcription in cancer cells, and its cytoplasmic location in breast cancer is associated with elevated expression of tumor cell hypoxia-inducible factor 1 alpha. Cancer Res 64:6075–6081 15342390 10.1158/0008-5472.CAN-04-0708

[CR15] Freedman SJ, Sun ZYJ, Kung AL, France DS, Wagner G, Eck MJ (2003) Structural basis for negative regulation of hypoxia-inducible factor-1 alpha by CITED2. Nat Struct Biol 10:504–512 12778114 10.1038/nsb936

[CR16] Gao H, Guan M, Sun Z, Bai C (2015) High c-Met expression is a negative prognostic marker for colorectal cancer: a meta-analysis. Tumour Biol 36:515–520 25636446 10.1007/s13277-014-2659-5

[CR17] Gong X, Carmon KS, Lin QS, Thomas A, Yi J, Liu QY (2012) LGR6 Is a high affinity receptor of R-spondins and potentially functions as a tumor suppressor. PLoS One 7:e37137 22615920 10.1371/journal.pone.0037137 PMC3355120

[CR18] Hartsock A, Nelson WJ (2008) Adherens and tight junctions: structure, function and connections to the actin cytoskeleton. Biochim Biophys Acta Biomembr 1778:660–669 10.1016/j.bbamem.2007.07.012 PMC2682436 17854762

[CR19] Havenith MG, Arends JW, Simon R, Volovics A, Wiggers T, Bosman FT (1988) Type-IV collagen immunoreactivity in colorectal-cancer—prognostic value of basement-membrane deposition. Cancer 62:2207–2211 3179934 10.1002/1097-0142(19881115)62:10<2207::aid-cncr2820621023>3.0.co;2-l

[CR20] Huang DW, Sherman BT, Lempicki RA (2009a) Systematic and integrative analysis of large gene lists using DAVID bioinformatics resources. Nat Protoc 4:44–57 19131956 10.1038/nprot.2008.211

[CR21] Huang DW, Sherman BT, Lempicki RA (2009b) Bioinformatics enrichment tools: paths toward the comprehensive functional analysis of large gene lists. Nucleic Acids Res 37:1–13 19033363 10.1093/nar/gkn923 PMC2615629

[CR22] Huang KT, Takano EA, Mikeska T, Byrne DJ, Dobrovic A, Fox SB (2011) Aberrant DNA methylation but not mutation of CITED4 is associated with alteration of HIF-regulated genes in breast cancer. Breast Cancer Res Treat 130:319–329 21755341 10.1007/s10549-011-1657-1

[CR23] Jiang W, Hiscox S, Matsumoto K, Nakamura T (1999) Hepatocyte growth factor scatter factor, its molecular, cellular and clinical implications in cancer. Crit Rev Oncol Hematol 29:209–248 10226727 10.1016/s1040-8428(98)00019-5

[CR24] Jorissen RN, Gibbs P, Christie M, Prakash S, Lipton L, Desai J, Kerr D, Aaltonen LA, Arango D, Kruhoffer M, Orntoft TF, Andersen CL, Gruidl M, Kamath VP, Eschrich S, Yeatman TJ, Sieber OM (2009) Metastasis-associated gene expression changes predict poor outcomes in patients with Dukes stage B and C colorectal cancer. Clin Cancer Res 15:7642–7651 19996206 10.1158/1078-0432.CCR-09-1431 PMC2920750

[CR25] Kallio MA, Tuimala JT, Hupponen T, Klemela P, Gentile M, Scheinin I, Koski M, Kaki J, Korpelainen EI (2011) Chipster: user-friendly analysis software for microarray and other high-throughput data. BMC Genom 12:507 10.1186/1471-2164-12-507 PMC3215701 21999641

[CR26] Kuhn S, Koch M, Nubel T, Ladwein M, Antolovic D, Klingbeil P, Hildebrand D, Moldenhauer G, Langbein L, Franke WW, Weitz J, Zoller M (2007) A complex of EpCAM, claudin-7, CD44 variant isoforms, and tetraspanins promotes colorectal cancer progression. Mol Cancer Res 5:553–567 17579117 10.1158/1541-7786.MCR-06-0384

[CR27] Leibovitz A, Stinson JC, McCombs WB III, McCoy CE, Mazur KC, Mabry ND (1976) Classification of human colorectal adenocarcinoma cell lines. Cancer Res 36:4562–4569 1000501

[CR28] Liu Y, Yu XF, Zou J, Luo ZH (2015) Prognostic value of c-Met in colorectal cancer: a meta-analysis. World J Gastroenterol 21:3706–3710 25834339 10.3748/wjg.v21.i12.3706 PMC4375596

[CR29] Lum AM, Wang BB, Beck-Engeser GB, Li L, Channa N, Wabl M (2010) Orphan receptor GPR110, an oncogene overexpressed in lung and prostate cancer. BMC Cancer 10:40 20149256 10.1186/1471-2407-10-40 PMC2830182

[CR30] Martin TA, Harrison G, Mansel RE, Jiang WG (2003) The role of the CD44/ezrin complex in cancer metastasis. Crit Rev Oncol Hematol 46:165–186 12711360 10.1016/s1040-8428(02)00172-5

[CR31] Mees ST, Mennigen R, Spieker T, Rijcken E, Senninger N, Haier J, Bruewer M (2009) Expression of tight and adherens junction proteins in ulcerative colitis associated colorectal carcinoma: upregulation of claudin-1, claudin-3, claudin-4, and beta-catenin. Int J Colorectal Dis 24:361–368 19184060 10.1007/s00384-009-0653-y

[CR32] Meniel V, Song F, Phesse T, Young M, Poetz O, Parry L, Jenkins JR, Williams GT, Dunwoodie SL, Watson A, Clarke AR (2013) Cited1 deficiency suppresses intestinal tumorigenesis. PLoS Genetics 9:1003638 10.1371/journal.pgen.1003638 PMC3731217 23935526

[CR33] Moffat J, Grueneberg DA, Yang X, Kim SY, Kloepfer AM, Hinkle G, Piqani B, Eisenhaure TM, Luo B, Grenier JK, Carpenter AE, Foo SY, Stewart SA, Stockwell BR, Hacohen N, Hahn WC, Lander ES, Sabatini DM, Root DE (2006) A lentiviral RNAi library for human and mouse genes applied to an arrayed viral high-content screen. Cell 124:1283–1298 16564017 10.1016/j.cell.2006.01.040

[CR34] Nakayama F, Semba S, Usami Y, Chiba H, Sawada N, Yokozaki H (2008) Hypermethylation-modulated downregulation of claudin-7 expression promotes the progression of colorectal carcinoma. Pathobiology 75:177–185 18550915 10.1159/000124978

[CR35] Nasu T, Oku Y, Takifuji K, Hotta T, Yokoyama S, Matsuda K, Tamura K, Ieda J, Yamamoto N, Takemura S, Nakamura Y, Yamaue H (2013) Predicting lymph node metastasis in early colorectal cancer using the CITED1 expression. J Surg Res 185:136–142 23746764 10.1016/j.jss.2013.05.041

[CR36] Orian-Rousseau V, Chen LF, Sleeman JP, Herrlich P, Ponta H (2002) CD44 is required for two consecutive steps in HGF/c-met signaling. Genes Develop 16:3074–3086 12464636 10.1101/gad.242602 PMC187488

[CR37] Oshima T, Kunisaki C, Yoshihara K, Yamada R, Yamamoto N, Sato T, Makino H, Yamagishi S, Nagano Y, Fujii S, Shiozawa M, Akaike M, Wada N, Rino Y, Masuda M, Tanaka K, Imada T (2008) Reduced expression of the claudin-7 gene correlates with venous invasion and liver metastasis in colorectal cancer. Oncol Rep 19:953–959 18357381

[CR38] Patara M, Santos EMM, Coudry RD, Soares FA, Ferreira FO, Rossi BM (2011) Ezrin expression as a prognostic marker in colorectal adenocarcinoma. Pathol Oncol Res 17:827–833 21465252 10.1007/s12253-011-9389-4

[CR39] Pennacchietti S, Michieli P, Galluzzo M, Mazzone M, Giordano S, Comoglio PM (2003) Hypoxia promotes invasive growth by transcriptional activation of the met protooncogene. Cancer Cell 3:347–361 12726861 10.1016/s1535-6108(03)00085-0

[CR40] Saotome I, Curto M, McClatchey AI (2004) Ezrin is essential for epithelial organization and villus morphogenesis in the developing intestine. Dev Cell 6:855–864 15177033 10.1016/j.devcel.2004.05.007

[CR41] Schuebel KE, Chen W, Cope L, Glockner SC, Suzuki H, Yi JM, Chan TA, Van Neste L, Van Criekinge W, van den Bosch S, van Engeland M, Ting AH, Jair K, Yu W, Toyota M, Imai K, Ahuja N, Herman JG, Baylin SB (2007) Comparing the DNA hypermethylome with gene mutations in human colorectal cancer. PLoS Genet 3:1709–1723 17892325 10.1371/journal.pgen.0030157 PMC1988850

[CR42] Sjoblom T, Jones S, Wood LD, Parsons DW, Lin J, Barber TD, Mandelker D, Leary RJ, Ptak J, Silliman N, Szabo S, Buckhaults P, Farrell C, Meeh P, Markowitz SD, Willis J, Dawson D, Willson JKV, Gazdar AF, Hartigan J, Wu L, Liu CS, Parmigiani G, Park BH, Bachman KE, Papadopoulos N, Vogelstein B, Kinzler KW, Velculescu VE (2006) The consensus coding sequences of human breast and colorectal cancers. Science 314:268–274 16959974 10.1126/science.1133427

[CR43] Tatum R, Zhang Y, Salleng K, Lu Z, Lin JJ, Lu Q, Jeansonne BG, Ding L, Chen YH (2010) Renal salt wasting and chronic dehydration in claudin-7-deficient mice. Am J Physiol Renal Physiol 298:F24–F34 19759267 10.1152/ajprenal.00450.2009 PMC2806124

[CR44] Tews B, Felsberg J, Hartmann C, Kunitz A, Hahn M, Toedt G, Neben K, Hummerich L, von Deimling A, Reifenberger G, Lichter P (2006) Identification of novel oligodendroglioma-associated candidate tumor suppressor genes in 1p36 and 19q13 using microarray-based expression profiling. Int J Cancer 119:792–800 16550607 10.1002/ijc.21901

[CR45] Tews B, Roerig P, Hartmann C, Hahn M, Felsberg J, Blaschke B, Sabel M, Kunitz A, Toedt G, Neben K, Benner A, von Deimling A, Reifenberger G, Lichter P (2007) Hypermethylation and transcriptional downregulation of the CITED4 gene at 1p34.2 in oligodendroglial tumours with allelic losses on 1p and 19q. Oncogene 26:5010–5016 17311001 10.1038/sj.onc.1210297

[CR46] Tomasini-Johansson BR, Sundberg C, Lindmark G, Gailit JO, Rubin K (1994) Vitronectin in colorectal adenocarcinoma—synthesis by stromal cells in culture. Exp Cell Res 214:303–312 7521845 10.1006/excr.1994.1262

[CR47] Torres-Martin M, Franco-Hernandez C, Martinez-Glez V, de Campos JM, Isla A, Casartelli C, Rey JA (2008) Mutational analysis of the CITED4 gene in glioblastomas. Cancer Genet Cytogenet 185:114–116 18722883 10.1016/j.cancergencyto.2008.05.013

[CR48] Turksen K, Troy TC (2011) Junctions gone bad: claudins and loss of the barrier in cancer. Biochim Biophys Acta 1816:73–79 21515339 10.1016/j.bbcan.2011.04.001

[CR49] Ueda J, Semba S, Chiba H, Sawada N, Seo Y, Kasuga M, Yokozaki H (2007) Heterogeneous expression of claudin-4 in human colorectal cancer: decreased claudin-4 expression at the invasive front correlates cancer invasion and metastasis. Pathobiology 74:32–41 17496431 10.1159/000101049

[CR50] van der Horst E, Chinn L, Wang M, Velilla T, Tran H, Madrona Y, Lam A, Ji M, Hoey TC, Sato AK (2009) Discovery of fully human anti-MET monoclonal antibodies with antitumor activity against colon cancer tumor models in vivo. Neoplasia 11:355–364 19308290 10.1593/neo.81536 PMC2657888

[CR51] Yahata T, Takedatsu H, Dunwoodie SL, Braganca J, Swingler T, Withington SL, Hur J, Coser KR, Isselbacher KJ, Bhattacharya S, Shioda T (2002) Cloning of mouse cited4, a member of the CITED family p300/CBP-binding transcriptional coactivators: induced expression in mammary epithelial cells. Genomics 80:601–613 12504852 10.1006/geno.2002.7005

[CR52] Yang ZL, Huo LJ, Chen H, Ni BB, Xiang J, Kang L, Wang L, Peng JS, Yuan YF, Wang JP (2013) Hypermethylation and prognostic implication of Syk gene in human colorectal cancer. Med Oncol 30:586 23609194 10.1007/s12032-013-0586-8

[CR53] Yoshida T, Sekine T, Aisaki K, Mikami T, Kanno J, Okayasu I (2011) CITED2 is activated in ulcerative colitis and induces p53-dependent apoptosis in response to butyric acid. J Gastroenterol 46:339–349 21165656 10.1007/s00535-010-0355-9

[CR54] Zeng ZS, Cohen AM, Guillem JG (1999) Loss of basement membrane type IV collagen is associated with increased expression of metalloproteinases 2 and 9 (MMP-2 and MMP-9) during human colorectal tumorigenesis. Carcinogenesis 20:749–755 10334190 10.1093/carcin/20.5.749

