# Supplementary figures and images for: CITED4 gene silencing in colorectal cancer cells modulates adherens/tight junction gene expression and reduces cell proliferation
Source: J Cancer Res Clin Oncol. 2015 Aug 5;142(1):225–37. doi: 10.1007/s00432-015-2011-5 (PMC4705123; doi:10.1007/s00432-015-2011-5)

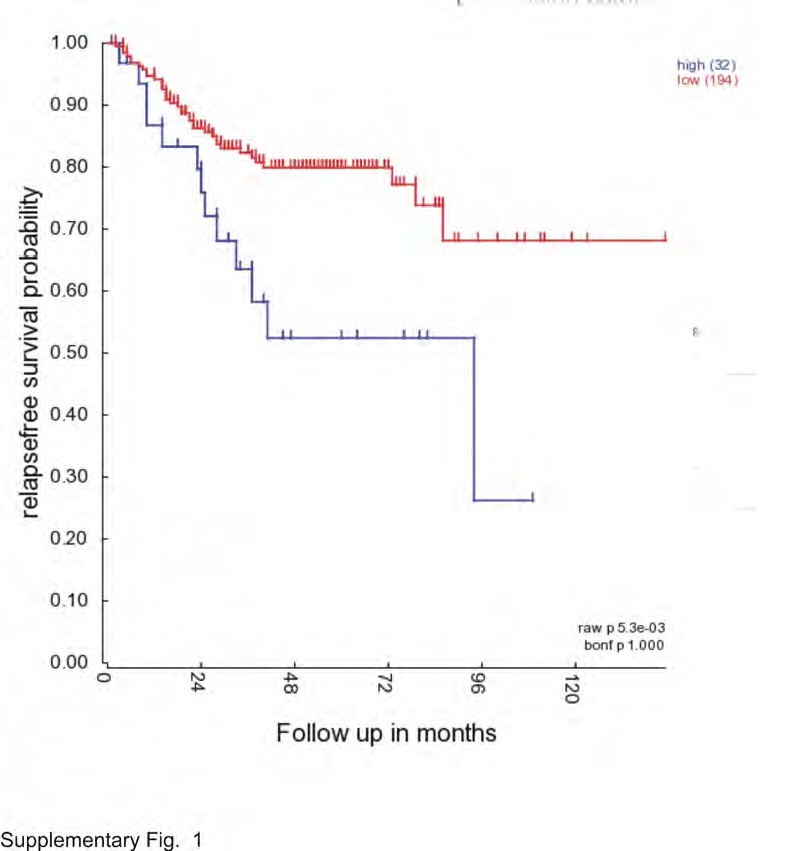

Supplement: Supplementary file 1 — Supplementary Fig. 1. Kaplan-Meier analysis of the data from Jorissen et al (Jorissen et al. 2009) obtained from the R2 database. Blue: patients with higher CITED4 expression; Red: patients with lower expression. Tick marks indicate patient censoring. (JPEG 68 kb) [file 432_2015_2011_MOESM1_ESM.jpg]

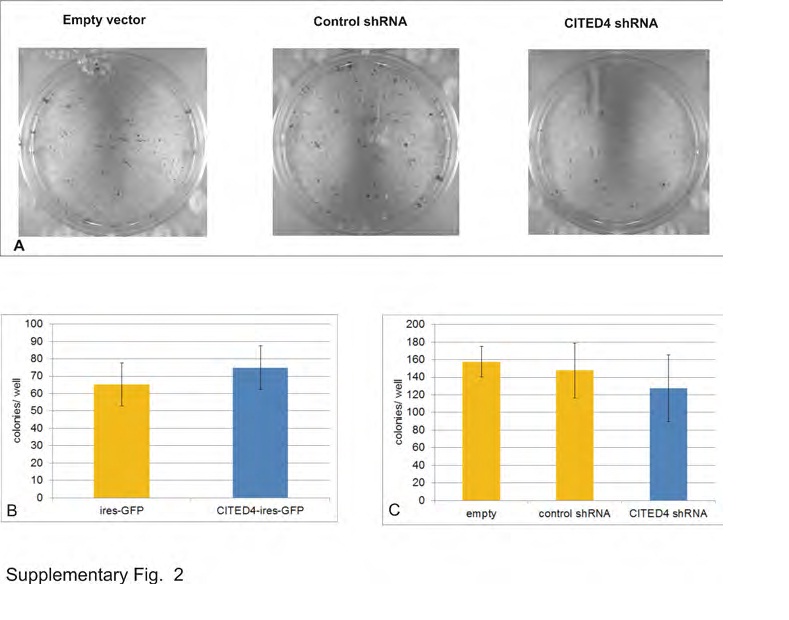

Supplement: Supplementary file 2 — Supplementary Fig. 2. Colony forming assays CITED4 overexpressing/shRNA knockdown cell lines. A Representative plates taken from the CITED4 shRNA analysis. B-C Evaluation of CITED4 overexpressing-B and shRNA knockdown C cell lines (2 experiments, n = 6). (JPEG 86 kb) [file 432_2015_2011_MOESM2_ESM.jpg]

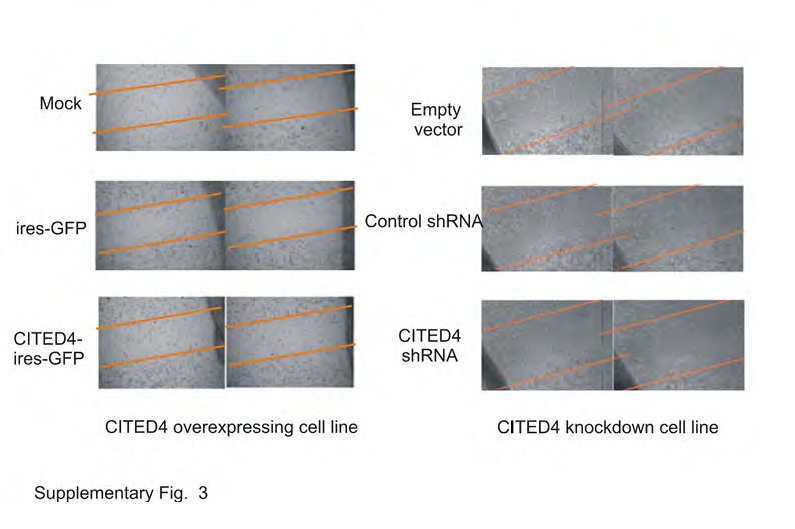

Supplement: Supplementary file 3 — Supplementary Fig. 3. Migration (scratch) assays-CITED4 overexpressing- (left) and shRNA-knockdown (right) cell line at 30 h after scratching. Two experiments, n = 6. (JPEG 80 kb) [file 432_2015_2011_MOESM3_ESM.jpg]

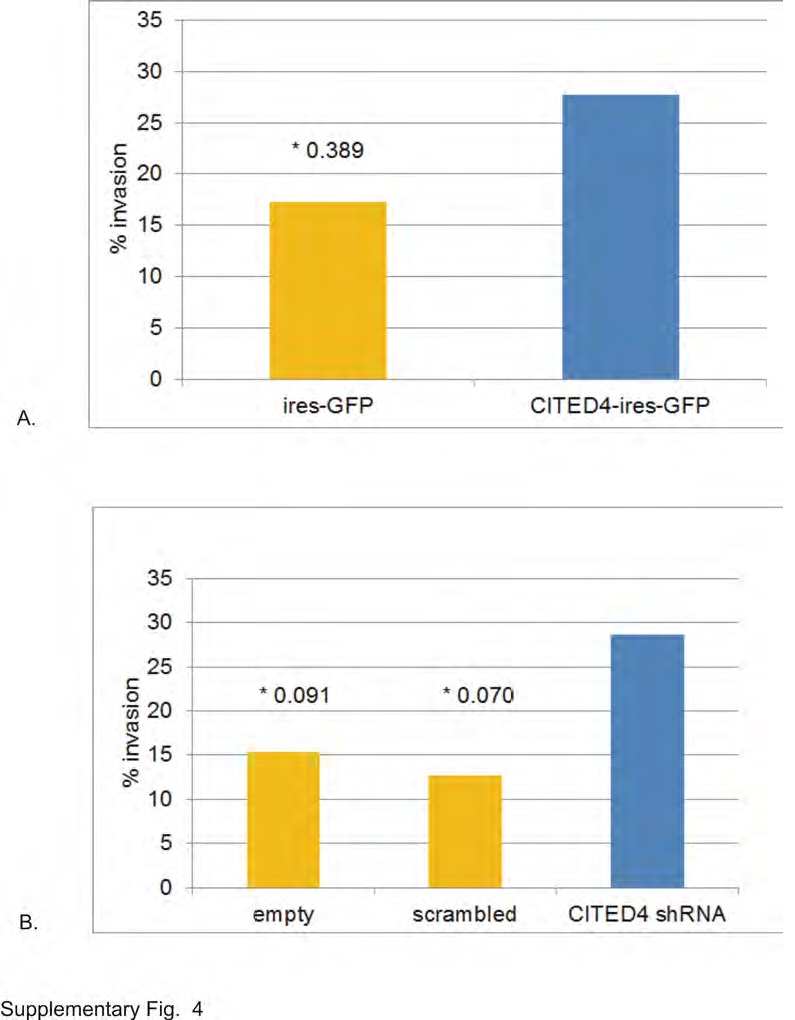

Supplement: Supplementary file 4 — Supplementary Fig. 4. Invasion assays. A CITED4 overexpressing permanent cell line and control. B CITED4 shRNA knockdown permanent cell line and controls. Invasion was measured 40 h after plating into transwells. *, t test values CITED4 overexpressing/shRNA knockdown cell lines vs. controls. Two experiments, n = 6. (JPEG 109 kb) [file 432_2015_2011_MOESM4_ESM.jpg]

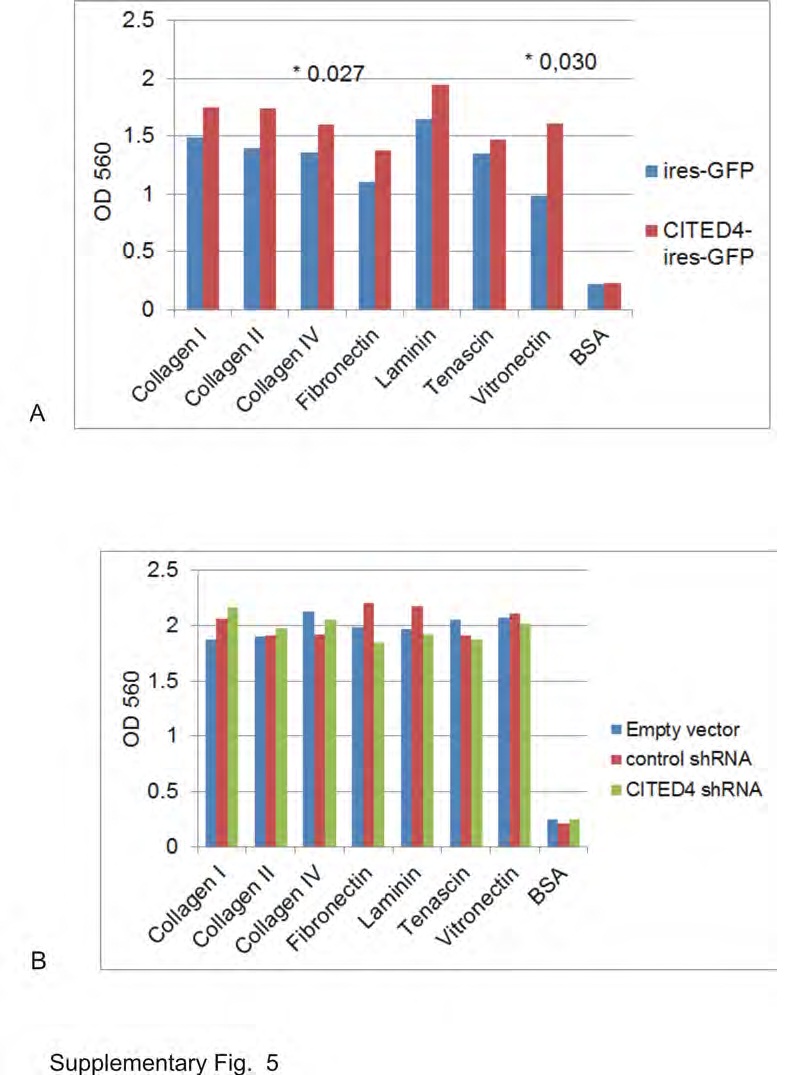

Supplement: Supplementary file 5 — Supplementary Fig. 5. Adhesion array analysis. A CITED4 overexpressing permanent cell line and control. B CITED4 shRNA knockdown permanent cell line and controls. *, Significant t test values (p < 0.05) CITED4 overexpressing/shRNA knockdown cell lines vs control. Two experiments, n = 6 (JPEG 150 kb) [file 432_2015_2011_MOESM5_ESM.jpg]

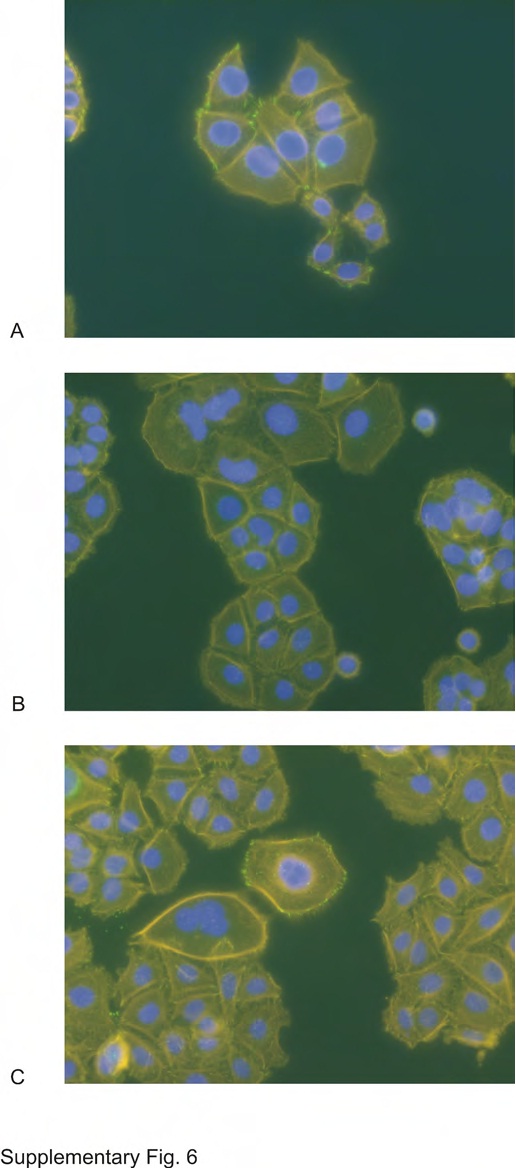

Supplement: Supplementary file 6 — Supplementary Fig. 6. F-actin (phalloidin) and vinculin immunofluorescence microscopy of the CITED4 shRNA permanent cell line and controls. A empty vector permanent cell line. B control shRNA permanent cell line. C CITED4 shRNA permanent cell line. Orange: actin (phalloidin) staining, green: vinculin staining, blue: DAPI. All pictures at 400x enlargement. (JPEG 99 kb) [file 432_2015_2011_MOESM6_ESM.jpg]
