# Supplementary material for: CITED4 gene silencing in colorectal cancer cells modulates adherens/tight junction gene expression and reduces cell proliferation
Source: J Cancer Res Clin Oncol. 2015 Aug 5;142(1):225–37. doi: 10.1007/s00432-015-2011-5 (PMC4705123; doi:10.1007/s00432-015-2011-5)
Supplement: Supplementary file 7 — Supplementary methods. (PDF 182 kb) [file 432_2015_2011_MOESM7_ESM.pdf]

Supplementary Methods

Migration assays: Initially, 2 x 10^6 cells were plated into 6 well plates. At day 1 the confluent cell layer was scratched with a one ml pipette. The medium was then changed and the scratched area was photographed at 0, 6, 24 and 30 hrs using a Leica DM-IRBE invert microscope (Leica, Wetzlar, Germany).

Invasion assays: Invasion assays of the overexpressing/ knockdown cell lines were performed using 24 well Biocoat Matrigel coated invasion assay chambers, as well as control chambers, according to the manufacturer's instructions (BD, Heidelberg, Germany). The assay was optimized for incubation time and cell number. Briefly, 1 x 10^6 cells were grown in RPMI1640 plus 10% FCS and penicillin/ streptomycin for 3 days. The cells were then incubated in serum free RPMI-1640 medium overnight. The cells were then detached by incubation with 5mM EDTA/ PBS for 15 min by 37° C, taken up in serum free medium, washed once in PBS, counted, and then taken up in in RPMI-1640/ 1% BSA at a concentration of 2 x 10^5 cells/ ml. 500 µl of the cell suspension was added to each pre-wetted lower control/ matrigel chambers, containing RPMI160 and 10% FCS as a chemo-attractant, and incubated for 40 hrs at 37° C. Thereafter, the medium in the upper chamber was removed and the upper membrane scrubbed with a cotton swab in order to remove non-migrating cells. The cells which migrated through the chamber membrane were washed 1x in PBS, fixed in methanol for 2 minutes, stained for 7 minutes using 0.2% crystal violet/ 10% methanol, destained in distilled water and the membrane mounted on microscope slides. The number of invading cells was determined by manual counting of multiple photographic images taken from each membrane using the ImageJ program. Percent invasion was defined as the percentage of cells migrating through the Matrigel containing membrane divided by the cells migrating through membranes without the Matrigel coating.

Cell cycle analysis: 5 x 10^4 cells were passaged into 12 well plates in RPMI-1640 plus 10% FCS and pencillin/ streptomycin. After 4 days, the medium was removed, the cells then trypsinized, taken up in serum containing medium, vortexed, washed in PBS and taken up in 1 ml cold 70% ethanol in PBS. The cells were fixed overnight at -20° C. Thereafter the cells were pelleted, washed 1x in PBS, then, taken up in 200 ul staining solution (0,1% Triton x100/ PBS, 0,2 mg/ml RNase A, 25,0 µg/ml propidium iodide). After incubation for 30 min in the dark, the labeled cells are analyzed using a BD-FACS CANTOII flow cytometer (Becton-Dickinson, Heidelberg, Germany).

Adhesion array assays: The adhesiveness of the overexpression/ knockdown cell lines to various protein substrates were analyzed using a ECM Cell Adhesion Array Kit-Colorimetric according to the manufacturers recommendations (Merck-Millipore, Billerica, MA), Briefly, 1 x 10^6 cells were plated into 10 cm dishes (10 ml RPM-1640I plus 10% FCS and penicillin/streptomycin) and grown at 37° C

and 5% CO_2. On the fifth day the cells were detached by incubation with 5 ml of 5mM EDTA in PBS for 15 min., inactivated using serum free RPMI-1640, counted and adjusted to 2 x 10^6 cells/ ml in assay buffer. 100 µl of this cell suspension was used for each assay. The cultures were incubated for 1 ½ hrs at 37° C. Thereafter, the cells were carefully removed, washed, stained with crystal violet, destained, and solubilized according to the manufacturer's instructions then quantitated photometrically (OD560) in a Mithras spectrophotometer.
