# Supplementary material for: CITED4 gene silencing in colorectal cancer cells modulates adherens/tight junction gene expression and reduces cell proliferation
Source: J Cancer Res Clin Oncol. 2015 Aug 5;142(1):225–37. doi: 10.1007/s00432-015-2011-5 (PMC4705123; doi:10.1007/s00432-015-2011-5)
Supplement: Supplementary file 8 — Supplementary Table 1. Oligonucleotide primers for cloning and qRT-PCR analysis. (PDF 215 kb) [file 432_2015_2011_MOESM8_ESM.pdf]

Supplementary Table 1 Primer pairs for qRT-PCR analysis and CITED4 cloning

Primers for cloning of CITED4 open reading frame				
Gene	RefSeq designations	Size (bp)	Annealing temp. (C)	Sequence
CITED4 (ORF)	nm_133467	556	72	atggccgaccacctgatgctc<br>tcagcgctcacggagccggc
Primers for Taqman analysis				
Gene	RefSeq	Size (bp)	Annealing temp. (C)	Sequence
CITED4	nm_133467	83	68	atgctcgccgagggctaccgc<br>ggcagagtccggagcgcatga
SYK	nm_001135052	139	57	gatcaggcccaaggaggtttac<br>tattttcacagccacggttttc
GPR64	nm_001079860	149	59	cctgcgaatgtcaacactacca<br>atcatttctcctgcgaggttag
GPR110	nm_153840	134	55	aaacatctaggcccagtcgaag<br>ctaattagcccatgtgaccata
LGR6	nm_021636	132	58	gaactggggttccataacaaca<br>caggtactggaatgccgatctt
IER5	nm_016545	102	61	aacttagagcagccgccgagtg<br>cgagaaactggaaccgaagatg
CLDN7	nm_001307	81	56	atgagagcaaggctgggtaccg<br>agatcccaggtcacacatactc
MET	nm_000245	99	58	tttcaaatggccacgggacaac<br>tgccaccagccataggaccgta
ß-CATENIN	nm_001904	148	59	gagcagggtgccattccacgac<br>ccggtacaaccttcaactattt
EZRIN	nm_003379	102	60	ggtactttggcctccactatgt<br>ggtagaacttggcccggaactt
ANK3	nm_001149	107	55	cacgggacagcatgatgattga<br>agcccagctgtaatgtctaaga
AMOTL1	nm_130847	137	57	aacagaggagaaccgggtgctt<br>ctcgcttggtggtagacttgac
GPR56	nm_201524	118	61	agtctgctcttcctggtccaag<br>ggtctggtgtgggtttgtagtg
DCTN2	nm_006400	103	59	gtggggacaaagggacttgatt<br>cccagaccctctccaagcatct
PGK1	nm_000291	71	50	aagtgaagctcggaaagcttctat<br>agggaaaagatgcttctggg
