# Supplementary material for: CITED4 gene silencing in colorectal cancer cells modulates adherens/tight junction gene expression and reduces cell proliferation
Source: J Cancer Res Clin Oncol. 2015 Aug 5;142(1):225–37. doi: 10.1007/s00432-015-2011-5 (PMC4705123; doi:10.1007/s00432-015-2011-5)
Supplement: Supplementary file 9 — Supplementary Table 2. Antibodies used for western blot analysis and fluorescence microscopy. (PDF 181 kb) [file 432_2015_2011_MOESM9_ESM.pdf]

Supplementary Table 2 Antibodies and labeled proteins used for immunofluorescence microscopy

<table>
<tr><td colspan="5">Western blotting</td></tr>
<tr><td>Gene</td><td>Antibody name</td><td>Dilution WB</td><td>secondary antibody (WB)</td><td>Dilution (WB)</td></tr>
<tr><td>CITED4</td><td>TA307013 (Origene)</td><td>1:500</td><td rowspan="4">Anti-rabbit HRP, Cell Signaling Technologies 7074S</td><td>1:1000</td></tr>
<tr><td>GPR110</td><td>AB75306 (Abcam)</td><td>1:500</td><td>1:1000</td></tr>
<tr><td>LGR6</td><td>L4169 (Sigma)</td><td>1:1000</td><td>1:1000</td></tr>
<tr><td>CLDN7</td><td>PAS-23689 (Thermo)</td><td>1:500</td><td>1:1000</td></tr>
<tr><td>EZRIN</td><td>MA5-13862 (Thermo)</td><td>1:1000</td><td rowspan="3">Anti-mouse HRP, Cell Signaling Technologies 7076S</td><td>1:1000</td></tr>
<tr><td>SYK</td><td>4D10.1 (Millipore)</td><td>1:1000</td><td>1:1000</td></tr>
<tr><td>GAPDH</td><td>CB1001 (Millipore)</td><td>1:10000</td><td>1:1000</td></tr>
<tr><td colspan="5">Fluorescence microscopy</td></tr>
<tr><td>Gene</td><td>Antibody name</td><td>Dilution IIF</td><td>Secondary antibody IIF</td><td>Dilution IIF</td></tr>
<tr><td>VINCULIN</td><td>90227 (Millipore)</td><td>1:200</td><td>Anti-mouse Alexa488 Molecular Probes A21204</td><td>1:200</td></tr>
<tr><td>F-ACTIN (TRIC-Phalloidin)</td><td>90228 (Millipore)</td><td>1:200</td><td>-</td><td>-</td></tr>
</table>
